# Summer versus winter: the impact of the seasons on oocyte quality in in vitro fertilization cycles

**DOI:** 10.1590/1806-9282.20240408

**Published:** 2024-09-16

**Authors:** Regis Yukio Cho, Mariana Mitiko Aseka, Kahisa Natiele Fontana Dal Toso, Arthur William Passos, Jaime Kulak, Vivian Ferreira do Amaral, Edward Araujo

**Affiliations:** 1 Universidade Federal do Paraná, Department of Obstetrics and Gynecology – Curitiba (PR), Brazil.; 2 Fertility Clinic, Androlab – Curitiba (PR), Brazil.; 3 Universidade Federal de São Paulo, Paulista School of Medicine, Department of Obstetrics – São Paulo (SP), Brazil.; 4 Universidade Municipal de São Caetano do Sul, Discipline of Woman Health – São Caetano do Sul (SP), Brazil.

**Keywords:** Assisted reproduction, Infertility, In vitro fertilization, Quality assurance

## Abstract

**OBJECTIVE:**

The aim of this study was to determine the effects of seasons (winter vs. summer) on oocyte quality in infertile women undergoing ovulation induction for in vitro fertilization.

**METHODS:**

This retrospective cross-sectional study assessed 155 cycles of in vitro fertilization-induced ovulation in women, with 71 and 84 cycles occurring in the summer and winter, respectively. Oocytes were evaluated for quality, with 788 and 713 assessed during summer and winter, and classified according to Nikiforov's categories: (a) category I, good quality; (b) category 2, medium quality; and (c) category 3, low quality.

**RESULTS:**

Thickened zona pellucida (p<0.001), increased perivitelline space (p<0.001), oocyte shape abnormalities (p=0.01), and the presence of refractile bodies (p<0.0001) were more frequent in the summer cycles, whereas cytoplasmic granularity (p<0.001) was more frequent in the winter cycles. In winter, we observed a higher frequency of category 3 (p<0.001) and category 2 (p<0.001) oocytes and a lower frequency of category 1 (p<0.001) oocytes.

**CONCLUSION:**

Oocyte dysmorphisms were found in 70–80% of cases and were more common in winter. The main features include a thickened zona pellucida, enlarged perivitelline space, irregular shape, and cytoplasmic granularity. This implies better-quality oocytes in the summer than in the winter. However, retrospective studies have limitations due to data collection biases and potential confounding variables such as diet and exercise. Future research is needed to confirm these findings and explore the underlying mechanisms.

## INTRODUCTION

According to the World Health Organization, infertility is a global public health problem affecting approximately 48 million couples and 186 million people (i.e., 10–15% of the world's population), of whom approximately 40 and 60% have primary and secondary infertility, respectively^1,2^. Assisted reproductive techniques (ARTs) are the main treatment for infertility; however, ART programs are limited to a small portion of the population owing to their high costs and low success rate of approximately 29.9%^3^. In vitro fertilization (IVF) is responsible for 2–5% of all newborns conceived today, and these rates tend to increase depending on the availability and coverage of this method by health insurance plans worldwide^4^.

In vitro fertilization pregnancy rates range from 30 to 70%, depending on the patient's age, body mass index (BMI), uterine and ovarian factors, stimulation dosing regimen and protocol, and use of fresh or frozen embryos. The interaction between these factors determines the oocyte numbers and pregnancy rates^5^. Seasonality is also a potential factor affecting fertilization rates, although no consensus has been reached yet in the literature^6,7^. The influence of season on naturally occurring conceptions (i.e., without ART) can be observed in birth rates^6-8^. This influence may be due to the effect of the season on intercourse frequency, ovulation rate, semen quality, postovulation oocyte quality, preimplantation conceptus, and endometrial receptivity^9^. Authors studying different climatic regions have found a significant influence of the seasons of the year on the results of ART procedures, with increased pregnancy rates observed during the summer^6,10^. Other authors have observed such an association in spring^6,9^.

Photoperiod is a major factor that causes seasonal variations in mammalian reproduction^11^. Changes in light intensity associated with seasonality alter the secretion of melatonin, a hormone produced at higher concentrations at night, and may be one of the factors that influence gonadal function, altering the rhythm of gonadotropin-releasing hormone secretion and increasing the number of mature oocytes, fertilization rates, and number of high-quality embryos^12^. Although there is no consensus in the literature regarding the seasonal factors responsible for variations in ART success rates, a trend in post-2019 studies has been observed to attribute these variations to higher average temperatures in warmer seasons, particularly on the days when ART procedures are performed^10,13^.

The quality of oocytes in IVF cycles is fundamental because embryonic development starts from this cellular structure and can be directly influenced by the season. Based on these considerations, we aimed to determine the effect of the season on oocyte quality in women with infertility undergoing ovulation induction for IVF.

## METHODS

This retrospective cross-sectional study included 155 infertile women aged 18–35 years who underwent IVF using intracytoplasmic sperm injection (ICSI) at an ART clinic and the Laboratory of Human Reproduction and Andrology (Androlab) in Curitiba City, southern Brazil. Overall, 1,501 oocytes were selected from 155 women undergoing ART, of whom 71 and 84 underwent the procedure in summer and winter, respectively, totaling 788 and 718 oocytes, respectively. This study was approved by the Ethics Committee of the Federal University of Paraná (UFPR) (No. 1.671.475/2.545.367).

Women undergoing IVF or oocyte freezing in winter and summer from 2017 to 2020 were included. To ensure a uniform participant, our study delineated an age range of 18–35 years and included only individuals who met the criteria for favorable oocyte quality, sufficient oocyte quantity, and normal ovarian reserve, as defined by both the Bologna and Poseidon criteria. We excluded patients with a history of low ovarian reserve, deterioration of oocyte quality due to pelvic radiation or chemotherapy, oophorectomy and/or oophoroplasty, or genetic disorders and those with thyroid disorders and hyperprolactinemia.

Each participant underwent ovulation induction with the administration of follicle-stimulating hormone and/or human menopausal gonadotropin on day 3 of their menstrual cycle, followed by ultrasound monitoring. When a follicular diameter of 14 mm was reached, pituitary suppression was initiated by administering a gonadotropin-releasing hormone antagonist. Subsequently, when a diameter of 18 mm was reached, urinary or human chorionic gonadotropin administration induced a luteinizing hormone peak. Oocyte retrieval was performed 35 h later. We limited our evaluation to oocyte quality to eliminate any potential bias from male factors on treatment outcomes. All oocytes were analyzed by a single embryologist who standardized the classification^14^.

Oocyte dysmorphology was classified into the following three categories according to Nikiforov et al.^15^:(1) category I or high-quality oocytes: oocytes with uniform cytoplasm, spherical or ovoid shape, medium round or ovoid first polar body, colorless zona pellucida (ZP), and small or absent perivitelline space (PVS); (2) category 2 or medium quality oocytes: oocytes with refractile bodies, fragmentation of the first polar body into two, dark ZP and other ZP abnormalities, large PVS, and debris in the PVS; and (3) category 3 or low-quality oocytes: oocytes with large singular cytoplasmic vacuoles or multiple small vacuoles, centrally located cytoplasmic granularity, and vacuoles of the smooth endoplasmic reticulum.

The Shapiro-Wilk test was used to assess the normality of the variables. In the exploratory analysis, a multivariate analysis was conducted to eliminate potential confounding factors. Age (years), weight (kg), height (cm), and BMI (kg/m^2^) were not normally distributed, whereas other variables followed a normal distribution. Normally distributed variables are presented as means and standard deviations, whereas non-normally distributed variables are presented as medians (25th and 75th percentiles). Differences between normal continuous variables were estimated using Fisher's exact test for abnormalities in oocyte size, cytoplasmic vacuoles, and smooth endoplasmic reticulum; for other normally distributed continuous variables, Pearson's chi-squared test with continuity correction was applied. For non-normally distributed variables, the Mann-Whitney U test was employed. All statistical analyses were performed using the Statistica 4.0 software (StatSoft Power Solutions, Inc., Palo Alto, CA, United States), and p-values <0.05 were considered statistically significant.

## RESULTS

Table 1 shows the characteristics of the participants according to the season in which ovulation was induced (summer vs. winter). The only differences between the two groups were the mean BMI (p=0.02) and rates of overweight and class I obesity (52.1 vs. 29.8%, respectively; p<0.001), which were slightly higher in women who underwent the procedure in the summer.

**Table 1:** Baseline characteristics of the women included in the study divided according to the season when ovulation was induced.

Baseline characteristics	Summer (n=71)(median [25–75%])*	Winter (n=84)(median [25–75%])*	p-value
Age (years)	32.0 (29.5–34.0)	32.0 (29.5–33.0)	0.79**
Weight (kg)	64.0 (59.0–73.5)	65.0 (58.0–71.5)	0.30**
Height (cm)	163.0 (159.0–167.0)	165.0 (160.5–168.0)	0.25**
Body mass index (kg/m^2^)	25.1 (22.2–28.0)	23.51 (21.8–25.3)	0.02**

* Median and 25–75th percentiles;

** Mann-Whitney U test.

Table 2 shows the data related to the 14 morphological dysmorphisms observed microscopically. A thickened ZP (p<0.001), increased PVS (p<0.001), oocyte shape abnormalities (p=0.01), and cytoplasmic granularity (p<0.001) were more frequent in cycles induced in winter, whereas refractile bodies (p<0.0001) were more frequent in cycles induced in summer.

**Table 2:** Morphological dysmorphisms in oocytes from cycles induced in summer (n=788) and winter (n=713).

Oocyte dysmorphisms	Summer (n=788) n (%)	Winter (n=713) n (%)	p-value
Thickened zona pellucida	642 (81.5)	619 (86.8)	<0.001*
Enlarged perivitelline space	420 (53.30)	464 (65.05)	<0.001*
Debris in the perivitelline space	135 (17.1)	102 (14.3)	0.13*
Oocyte shape abnormalities	39 (4.9)	57 (8.0)	0.01*
Oocyte color abnormalities	126 (16.0)	102 (14.3)	0.36*
Oocyte size abnormalities	8 (1.0)	3 (0.4)	0.23**
Polar body fragmentation	264 (33.5)	237 (33.2)	0.91*
Polar body size	81 (10.3)	72 (10.1)	0.90*
Polar body shape	268 (34.0)	234 (32.8)	0.62*
Cytoplasmic vacuoles	13 (1.6)	19 (2.7)	0.21**
Smooth endoplasmic reticulum	8 (1.0)	7 (1.1)	0.99**
Refractile bodies	70 (8.9)	20 (2.8)	<0.0001*
Cytoplasmic granularity	554 (70.3)	556 (77.98)	<0.001*
Dark central granulation	51 (6.5)	50 (7.0)	0.67*

* Chi-squared test with Yates continuity correction.

** Fisher’s exact test.

Based on the criteria of Nikiforov etal.^15^, we observed a higher frequency of category 3 oocytes (considering the morphological criterion of cytoplasmic granularity: 78.0 vs. 70.3%, p<0. 001), a higher frequency of category 2 oocytes (considering the frequency of thickened ZP; 81.5 vs. 86.0%, p<0.001), and a lower frequency of category 1 oocytes (approximately 13.0 vs. 19%, p<0.001).

**Figure 1:**
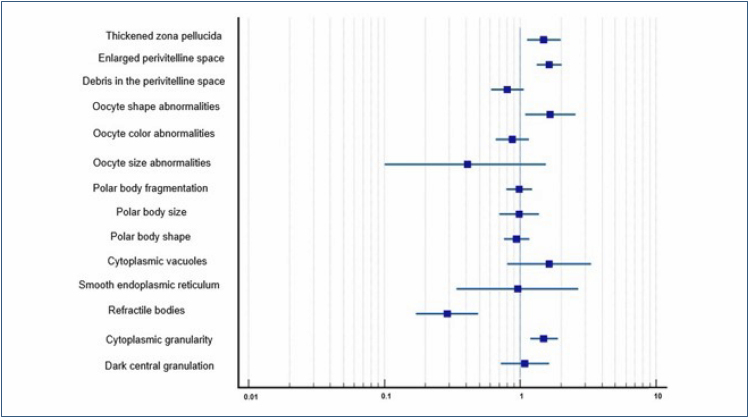
Odds ratio of the morphological changes found in 788 oocytes from cycles induced in summer and 713 oocytes from cycles induced in winter. Odds ratio: <1: summer; >1: winter.

Patients induced in winter compared to summer were more likely to have oocytes with thickened ZP (odds ratio [OR]=1.49, 95% confidence interval [CI]=1.12–1.98, p<0.01), enlarged PVS (OR=1.63, 95%CI=1.32–2.01, p<0.001), irregular shape (OR=1.66, 95%CI=1.09–2.54, p=0.01), and cytoplasmic granularity (OR=1.49, 95%CI=1.18–1.89, p<0.001), whereas those induced in summer compared to winter had greater OR of having oocytes with refractile bodies (OR=0.29, 95%CI=0.17–0.49, p<0.001) (Figure 1).

## DISCUSSION

The present study analyzed 1,501 oocytes from 155 women undergoing ART and found that dysmorphisms were present in approximately 70–80% of oocytes and were more frequent in procedures performed in winter than in summer. The main dysmorphisms observed were a thickened ZP, a large PVS, an irregular shape, and cytoplasmic granularity. The only dysmorphism found more frequently in summer than in winter was the presence of refractile bodies, which were present in<10% of oocytes. Overall, the frequency of good-quality oocytes was higher in the summer.

Since Brazil is a tropical country, we focused on winter versus summer to study the effect of seasons on oocyte dysmorphism, analyze the most contrasting seasons in terms of climatic variations, and obtain better observations in terms of temperature differences, light hours, and humidity, among other factors. The study location was also favorable, as it has better-defined seasons and pronounced temperature variations between seasons compared to other cities in Brazil. Our analysis covers several years (2017–2020) and reduces the possibility that an isolated factor in any season could directly affect the results. Overall, 70.2% of patients who underwent ovulation induction in winter had a BMI within normal limits, which confounded the analysis of a possible association between being overweight or obese and the finding of a higher proportion of oocyte abnormalities in winter.

Problems with the secretion or pattern of matrix glycoproteins may cause changes in the appearance of the ZP. Subtle changes in the three-dimensional structure of the ZP are more common than changes in the thickness or complete absence of the ZP. Notably, the inner ZP layer was highly organized and could be visualized using polarized light. Polarized light may also help predict the prognosis of embryos in terms of blastocyst formation and pregnancy^14^. Several studies have found no correlation between the ZP thickness and fertilization rates, pronuclear morphology, embryonic development, or clinical pregnancy^16,17^. Other studies have shown that approximately one-third of the oocytes have an enlarged PVS, a finding that is negatively associated with fertilization rate and embryo quality^14,16^. Studies have suggested that an enlarged PVS may be found in overmature oocytes, in which the oocyte has shrunk relative to the ZP and presents a large space in the surrounding zona^18,19^. The PVS can also enlarge when a large portion of the cytoplasm extrudes from the haploid chromosome during polar body formation^20^.

Severe dysmorphisms in the cytoplasmic texture may alter embryonic development and potential implantation; however, the biological significance of different degrees of ooplasmic heterogeneity remains unknown^21^. Current evidence suggests that a slightly heterogeneous cytoplasm may represent a variation in a normal oocyte during retrieval^15,20^. Cytoplasmic granularity is poorly defined in the literature and may depend on the calibration of the microscope used for the analysis. These morphological changes must be carefully distinguished from inclusions, such as refractile bodies, lipofuscin bodies, or organelle clusters^20^. This study did not show any correlation between the presence of these cytoplasmic inclusions and fertilization, embryo quality, or implantation rates^22^.

Studies have reported variable outcomes for oocytes with centralized granularity, including impaired pronuclear morphology and embryo quality after fertilization^23^, decreased survival, and impaired in vitro development of cryopreserved embryos^24^. One study found no association between central granularity and fertilization rates, embryo development, or pregnancy rates, although rates of continuing pregnancy (defined in the study as ≥20 weeks’ gestation) were severely compromised when embryos were transferred from oocytes with central granularity^25^.

A possible bias in the present study is the inclusion of patients who did not live in the city where the study was conducted (i.e., those who traveled to the study city to undergo the ART procedure), since the patients’ place of origin was not analyzed. Depending on the place of origin, the patient may not have been exposed to the seasonal variations analyzed in this study because the seasons are not well defined in several other Brazilian regions. The results of the present study indicate the need for further research on the influence of season on oocyte quality since the consensus on this topic is still lacking. Additionally, the study focused solely on winter and summer, potentially missing variations at other times of the year. There is no consensus among authors on this topic, underscoring the importance of comprehensive investigations that cover broader seasonal variations. This retrospective analysis serves as an initial step in addressing this gap in understanding and underscores the importance of future prospective studies to confirm and expand on these findings.

## CONCLUSION

In summary, oocyte dysmorphisms were present in approximately 70–80% of cases and were more frequent in the winter. The main dysmorphisms observed were a thickened ZP, an enlarged PVS, an irregular oocyte shape, and cytoplasmic granularity. These findings suggest that oocytes induced in the summer were higher in quality than those induced in the winter.
